# State-of-the-art performance of deep learning methods for pre-operative radiologic staging of colorectal cancer lymph node metastasis: a scoping review

**DOI:** 10.1136/bmjopen-2024-086896

**Published:** 2024-12-02

**Authors:** Benjamin Keel, Aaron Quyn, David Jayne, Samuel David Relton

**Affiliations:** 1 School of Computing, University of Leeds, Leeds, UK; 2 Leeds Teaching Hospitals NHS Trust, Leeds, UK; 3 Leeds Institute of Medical Research, University of Leeds, Leeds, UK; 4 Leeds Institute of Health Sciences, University of Leeds, Leeds, UK

**Keywords:** Gastrointestinal imaging, Gastrointestinal tumours, Machine Learning, Diagnostic Imaging, Magnetic resonance imaging, Review

## Abstract

**Abstract:**

**Objectives:**

To assess the current state-of-the-art in deep learning methods applied to pre-operative radiologic staging of colorectal cancer lymph node metastasis. Specifically, by evaluating the data, methodology and validation of existing work, as well as the current use of explainable AI in this fast-moving domain.

**Design:**

Scoping review.

**Data sources:**

Academic databases MEDLINE, Embase, Scopus, IEEE Xplore, Web of Science and Google Scholar were searched with a date range of 1 January 2018 to 1 February 2024.

**Eligibility criteria:**

Includes any English language research articles or conference papers published since 2018 which have applied deep learning methods for feature extraction and classification of colorectal cancer lymph nodes on pre-operative radiologic imaging.

**Data extraction and synthesis:**

Key results and characteristics for each included study were extracted using a shared template. A narrative synthesis was then conducted to qualitatively integrate and interpret these findings.

**Results:**

This scoping review covers 13 studies which met the inclusion criteria. The deep learning methods had an area under the curve score of 0.856 (0.796 to 0.916) for patient-level lymph node diagnosis and 0.904 (0.841 to 0.967) for individual lymph node assessment, given with a 95% confidence interval. Most studies have fundamental limitations including unrepresentative data, inadequate methodology, poor model validation and limited explainability techniques.

**Conclusions:**

Deep learning methods have demonstrated the potential for accurately diagnosing colorectal cancer lymph nodes using pre-operative radiologic imaging. However, several methodological and validation flaws such as selection bias and lack of external validation make it difficult to trust the results. This review has uncovered a research gap for robust, representative and explainable deep learning methods that are end-to-end from automatic lymph node detection to the diagnosis of lymph node metastasis.

**STRENGTHS AND LIMITATIONS OF THIS STUDY**

- This scoping review is reported in line with the Preferred Reporting Items for Systematic Reviews and Meta-Analyses extension for Scoping Reviews (PRISMA-ScR) guidelines, and was carried out according to the study protocol with well-defined research questions, search terms, inclusion and exclusion criteria and an extraction template for consistency.
- The search was conducted across five key databases, with snowball searching of the references of included studies and searches of Google Scholar; however, further studies may have been identified by including other academic databases.
- Only one person reviewed all the studies, which could introduce potential bias or oversight.
- The number of studies included was small, but this allowed for an in-depth discussion of each study, providing a full picture of the existing knowledge base for deep learning methods.

## Introduction

 Colorectal cancer (CRC) is one of the leading causes of cancer-related death worldwide. Despite noticeable improvement in early diagnosis and management, morbidity and mortality remain high, with a 5-year survival of 65%.^1^ One key prognostic factor for CRC is the involvement of lymph nodes (LNs). Accurate cancer staging including LN status remains the cornerstone of multidisciplinary team decision-making to ensure that patients are correctly identified for organ preserving or radical strategies including neo-adjuvant therapy to reduce local recurrence. Inaccuracies can lead to under or over treatment, impacting patient toxicity, morbidity and cancer-specific outcomes.^2 3^ However, clinical rules-based evaluation of LNs on pre-operative radiologic imaging has limited diagnostic sensitivity and specificity of 73% (95% confidence interval (CI): 68% to 77%) and 74% (68% to 80%), respectively.^4^

In recent years, machine learning algorithms have been applied to this task, aiming to improve the diagnostic accuracy of CRC lymph node metastasis (LNM). The two most popular approaches are deep learning methods and traditional machine learning algorithms in combination with radiomic feature representations. Deep learning is a subfield of machine learning where the algorithms are based around neural networks. Radiomics is a collection of methods that aim to extract quantitative features from medical images, which can be used with data-driven learning algorithms. These features typically include geometric size and shape characteristics, texture patterns, and statistics generated from the pixel intensity values. While deep learning methods are now state-of-the-art in many cancer imaging tasks,^5 6^ the key limitation and barrier to clinical adoption is that methods typically lack explainability and interpretability. The foundation of state-of-the-art models is usually a convolutional neural network (CNN), although in some areas they are outperformed by other methods such as vision transformers.^7^ These algorithms are black-box models which raises concerns about explaining diagnoses and how the model might perform ‘in the wild’ including on outlier/edge cases, in addition to concerns around model bias and fairness when using historical data. Some existing techniques such as Gradient-weighted Class Activation Mapping (Grad-CAM)^8^ aim to improve the explainability of CNN prediction tools by indicating the important pixels and regions of the image in a gradient heatmap. However, ultimately, the numerical representations of the images and the mechanics of neural network models are not interpretable. On the other hand, radiomics uses statistical and geometric features which are directly interpretable and often align with radiological concepts. Additionally, radiomic features may be included based on clinical insight and disease-specific knowledge.

The literature search found several review articles that had some overlap with our research questions; however, none specifically focused on deep learning methods. The most similar is a systematic review by Bedrikovetski *et al*,^9^ exploring the application of deep learning and radiomics in pre-operative CRC LNM prediction and comparing the performance of these machine learning models with radiologists. They found that for rectal cancer there was a per patient area under the curve (AUC) of 0.917 (0.882 to 0.952) for deep learning models, 0.808 (0.739 to 0.876) for radiomics and 0.688 (0.603 to 0.772) for radiologists. Note that these AUC scores were pooled across multiple studies and given with a 95% CI. Another highly relevant review was conducted by Peng *et al,*^10^ summarising the evidence and progress made for rectal cancer LNM prediction across multiple imaging modalities and biomarkers. They compared deep learning methods, radiomics with machine learning and clinical evaluation. The search was completed in December 2022, more recently than Bedrikovetski *et al*.^9^ These literature reviews have shown that radiomics is already well established and that these methods have been rigorously validated for this task. However, the classification performance potential of radiomics features may be lower than deep learning, and radiomics-based methods for CRC can have limited reproducibility due to inconsistencies in standardisation, imaging parameters and feature selection.^11 12^ Overall, radiomics offers a good baseline for diagnostic performance and can be useful for knowledge transfer of the best methodological and validation practices, therefore, the discussion will compare to a well-validated radiomics study. By comparison, deep learning approaches are relatively unexplored but have produced encouraging results. The previously mentioned reviews are limited to 2022, and deep learning approaches have advanced considerably since then, both in general and in this domain. First and foremost, this review aims to bridge this gap and assess the current state of the field, including the quality of the existing knowledge base. As such, a scoping review is sufficient to give a broad representation of the current status quo.

The primary research objective (1) and two secondary research questions are as follows:

1. What is the current state-of-the-art for deep learning-based methods applied to predict LNM in CRC using pre-operative radiologic imaging?
2. What are the common limitations in the methodology and validation of the deep learning approaches?
3. To what extent are the existing deep learning-based methods explainable and interpretable?

The overall purpose is to provide a summary of the current state-of-the-art deep learning performance in this field and to identify key gaps in the existing research.

## Methods

The literature search was conducted on the databases MEDLINE, Embase, Scopus, IEEE Xplore and Web of Science. Additionally, snowball searching of the reference lists was performed for each of the included studies, and non-systematic searches of Google Scholar. The following search terms were used to identify relevant studies from the full text: (“colorectal” or “colon” or “rectal”) and (“cancer”) and (“ai” or (“artificial” and “intelligence”) or (“deep” and “learning”) or (“machine” and “learning”)) and (“lymph” and “node”) and (“mr” or “mri” or “ct” or “radiology”)). Note that minor modifications were required to match the syntax of each database, and a full breakdown is available in online supplemental material 1. The inclusion and exclusion criteria are given in table 1.

**Table 1:** Inclusion and exclusion criteria for the literature review

Inclusion	Exclusion
Colorectal cancer (CRC)	Not CRC / other anatomical cancer locations
Lymph node metastasis (LNM) prediction	Not LNM
Deep learning (DL)	Radiomics with machine learning / statistics
Features learned via DL	Features derived from radiomics only
Pre-operative radiologic imaging MRI or CT	Post-operative histopathology
Available in English	Not available in English
Article or conference paper	Review or other publication types
Published 1 January 2018 to 1 February 2024	Published pre-2018

Included articles were initially selected based on a review of the title and abstracts, followed by a more in-depth review of the methods and results to confirm all the inclusion criteria were met. This search strategy was conducted on the five databases with the following limitations: publication date range of 1 January 2018 to 1 February 2024, available in English, and document type: article or conference paper. This search was limited to 2018 as research papers more than 6 years old are likely to be superseded due to the rapid and accelerating progress in computer vision resulting from increased computing capacity, data availability and method development.^13^ An example of evidence for this is the leaderboard for the ImageNet dataset, where accuracy has improved from 82.9% to 92.4% over the years 2018–2024.^14^ Additionally, the research published between 2010 and 2020 was covered systematically in Bedrikovetski *et al*.^9^ The protocol for this review is attached as online supplemental material 2, it outlines the background and methodology, including an extraction template which defines the information to be captured from each article.

### Patient and public involvement

None.

## Results

**Figure 1:**
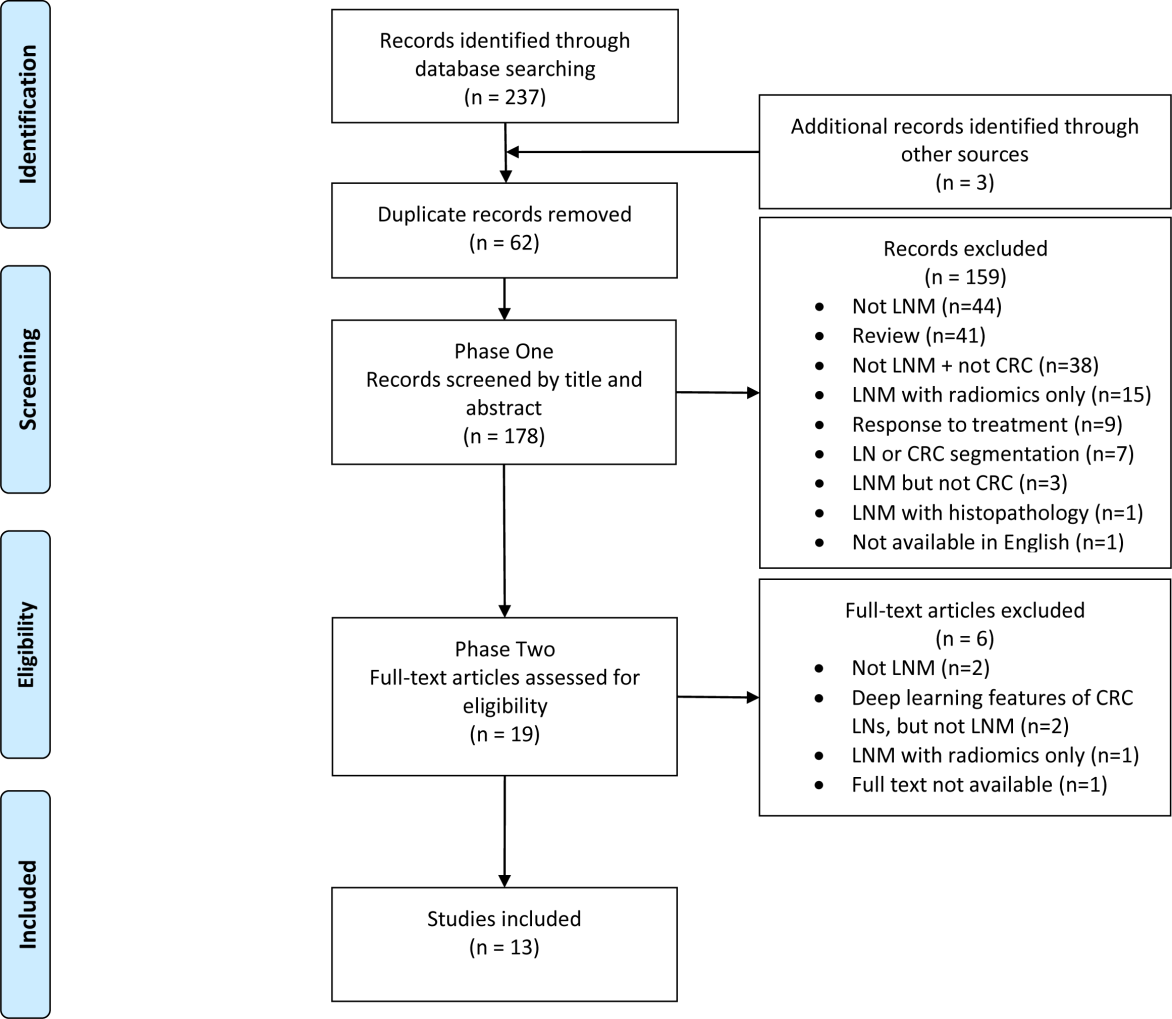
PRISMA-ScR diagram reporting document retrieval and selection. CRC, colorectal cancer; LNM, LN metastasis; LN, lymph node; PRISMA-ScR, Preferred Reporting Items for Systematic Reviews and Meta-Analyses extension for Scoping Reviews.

Of the 240 results collected, a total of 13 unique articles were included (5.4%). For robust and transparent reporting, this review follows the guidelines of the Preferred Reporting Items for Systematic Reviews and Meta-Analyses extension for Scoping Reviews (PRISMA-ScR).^15^ The PRISMA-ScR diagram is included in figure 1, providing a breakdown of the literature search and selection.

**Figure 2:**
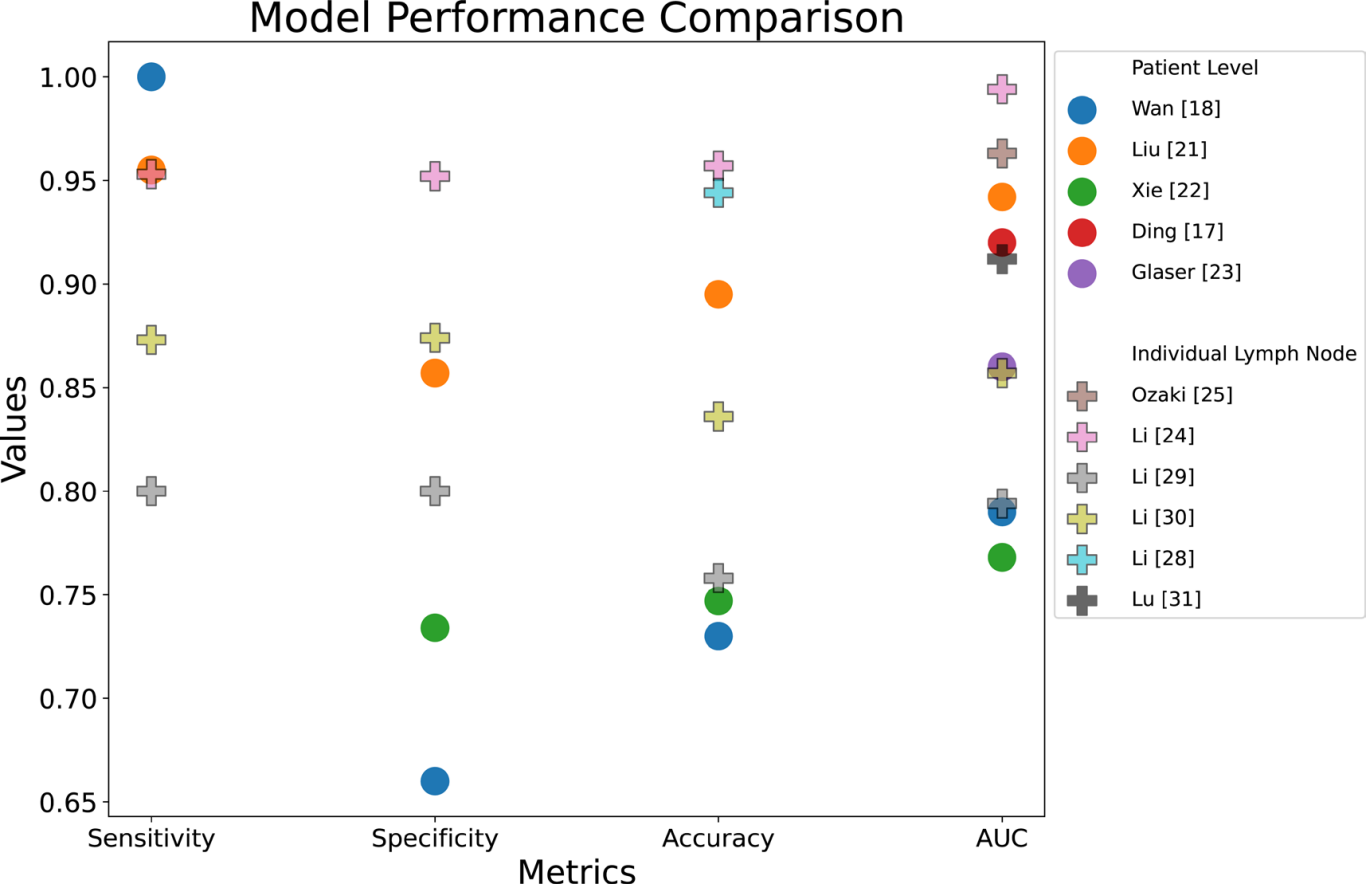
Performance metrics comparison graph. Note that the outlier result from Bedrikovetski *et al*^16^ has been excluded. AUC, area under the curve.

**Figure 3:**
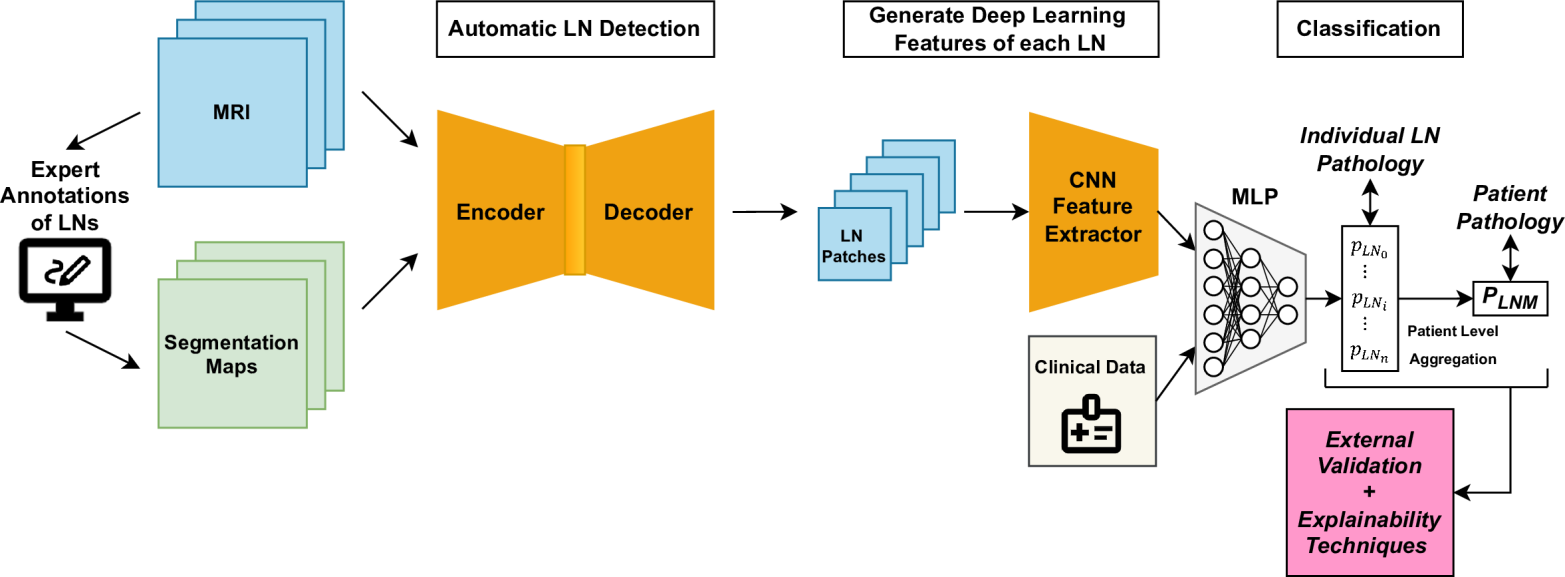
Recommended study design diagram presenting an automated detection and classification pipeline including a convolutional neural network (CNN) and multilayered perceptron (MLP) for both individual lymph node (LN) and patient-level lymph node metastasis (LNM) assessment.

Table 2 provides a summary of the included studies in this scoping review, including study details, a description of the data, model architecture, a key limitation and model performance (sensitivity, specificity, accuracy and AUC). These performance metrics are compared in figure 2. A visual representation of the recommended study design is shown in figure 3. This diagram integrates the best elements of existing approaches, serving as a basis for comparison and including all necessary components for a fully automated classification pipeline.

**Table 2:** Literature review summary of lymph node metastasis (LNM) prediction in CRC using deep learning, split by patient-level or individual LN assessment

Patient-level LN assessment							
Study details	Data summary	Model architecture	Key limitation	Sensitivity	Specificity	Accuracy	AUC
Bedrikovetski *et al*,^16^ 2023, Australia	1201 CT scans, CC, 500 scans with LN annotations	Shared ResNet-50 for segmentation and MLP classification	Non-expert annotations	0.966	0.052	37.4%	0.542
Wan *et al,*^18^ 2023, China	610 MRI scans, RC, *limited to T1–2,tumour annotations	LN level 2D and 3D ResNet with transfer learning and averaging predictions from logistic regression	Not using cross-validation	**1.000**	0.660	73.0%	0.790
Liu *et al,*^21^ 2023, China	282 CT scans, RC,Tumour annotations	ResNet-50, clinical and radiomics features, ML models and nomogram classifier	Test set of 57	0.955	**0.857**	**89.5%**	**0.942**
Xie *et al*,^22^ 2023, China	391 CT scans, CRC, LN annotations	LN and slice level ResNet-18 with an attention mechanism and logistic regression	No external validation		0.734	74.7%	0.768
Ding *et al*,^17^ 2020, China	545 MRI scans, RC, LN annotations	Faster R-CNN with transfer learning, clinical features, nomogram classifier and bounding box	153/183 in the test set had LNM				0.920
Glaser *et al*,^23^ 2020, Australia	123 CT scans, CC,595 individual LN annotations	DenseNet encoder-decoder with segmentation and MLP classifier	Test set of 23 patients				0.860

Individual LN assessment							
Study details	Data summary	Model architecture	Key limitation	Sensitivity	Specificity	Accuracy	AUC
Ozaki *et al*,^25^ 2023, Japan	3547 LNs on MRI, RC, *Includes patients who had chemoradiotherapy	ResNet-18 with transfer learning, logistic regression	Used same pathology label for pretreatment/post-treatment				0.963
Li *et al*,^24^ 2021, China	129 MRI scans, RC	Inception-v3 with transfer learning, logistic regression	Only included 1–2 LNs per patient	**0.953**	**0.952**	**95.7%**	**0.994**
Li *et al*,^29^ 2021, China	3364 MRI scans, CRC	LeNet/AlexNet with transfer learning, ML models and MLP	Used radiologist LNM diagnosis	0.800	0.800	75.8%	0.794
Li *et al*,^30^ 2021, China	3364 MRI scans, CRC	AlexNet with transfer learning and skip connections, MLP	Used radiologist LNM diagnosis	0.873	0.874	83.6%	0.857
Ding *et al*,^32^ 2019, China	414 MRI scans, RC	Faster R-CNN with transfer learning, MLP	All patients had LNM				
Li *et al*,^28^ 2018, China	619 LNs on MRI, CRC	Inception-v3 with transfer learning, MLP	Used radiologist LNM diagnosis			94.4%	
Lu *et al*,^31^ 2018, China	414 MRI scans, RC	Faster R-CNN with VVG16 and transfer learning, MLP and bounding box	All patients had LNM				0.912

Includes study details, a description of the data with the number of patients or number of LNs, image modality (MRI/CT), cancer location (colo (CRC) or (RC) or (CC)), and unique inclusion criteriaaspects marked with an asterisk. Also, a model architecture summary, a key limitation and model performance metrics: sensitivity, specificity, accuracy and area under the receiver operating characteristic curve (AUC) to three significant figures.. The best result for each metric is indicated in bold.

AUCarea under the curveCCcolon cancerCRCcolorectal cancerRCrectal cancer

### Current state-of-the-art deep learning-based methods for pre-operative radiologic prediction of LNM in CRC

The AUC scores with a 95% CI for the deep learning methods were found to be 0.856 (0.796 to 0.916) for patient-level models (not including the outlier result from Bedrikovetski *et al*^16^) and 0.904 (0.841 to 0.967) for diagnosis of specific LNs. In terms of the model architecture, all the included approaches use large CNNs (n=13), most of the time with pretrained weights for transfer learning (n=9), indicating that this is the current state-of-the-art approach. The most popular architectures include ResNet (n=5), Faster R-CNN (n=3), Inception-v3 (n=2) and AlexNet (n=2). Classification models used include multilayered perceptron (MLP) (n=7), logistic regression (single-layered MLP) (n=4) and nomograms (n=2). A limited number of studies included an automatic detection of the LNs (n=4). Lastly, several studies included a baseline comparison with radiologists (n=6), ablation studies or alternative deep learning architectures (n=5), and non-deep learning approaches (n=3). The approaches are split into patient-level and individual LN assessment.

#### Patient-level LN assessment

First, two studies used rectal cancer MRI scans.^17 18^ Ding *et al*^17^ built a nomogram LNM classifier and bounding box detector using the prediction from a Faster R-CNN model based on 545 rectal cancer MRI scans. Additionally, clinical variables were selected using regression analysis. The approach achieved an AUC of 0.92 and this was compared directly with radiologists. However, the testing set contained 153/183 patients with metastatic LNs which is unrepresentative of the target population. Wan *et al*^18^ implemented a patient-level model which averaged predictions from a logistic regression over multiple patches of an MRI, with a region of interest containing the primary tumour and the closest LNs for 610 patients with stage T1-2 rectal cancer. This is notable as they did not require expert segmentation of individual LNs. The approach used a large CNN architecture (ResNet) in 2D and 3D variations with weights pretrained on a medical dataset and achieved 0.79 AUC for the 3D method and 0.69 for 2D. The model was compared with three radiologists that had an average score of 0.54 AUC. This study is the first to assess LNM in early-stage rectal cancer which is a more challenging task due to the lower prevalence of LNM in stage T1-2, approximately 10%–26%.^19 20^

Finally, four approaches using CT scans are covered here.^1621^,^23^ Liu *et al*^21^ had a dataset of 282 rectal cancer CT scans with annotations of the primary tumour. They extracted deep learning features using a ResNet50 model as well as radiomic features and clinical data including the radiologic N stage. The radiomics and deep learning features were used to train models including a support vector machine (SVM), K-nearest neighbours and an MLP. The SVM model was selected and then included in a stacking nomogram which achieved 0.942 AUC, this model was further validated by comparing with radiologists. Xie *et al*^22^ implemented an attention-based multiple instance learning (MIL) framework for 391 CRC patients with CT scans. They used a ResNet-18 on the manually annotated individual LNs and the corresponding full 2D slices. Global-local cross-attention was implemented to fuse the features and then attention pooling was used to combine them into a patient-level bag representation. This approach also uses a nested-neural memory ordinary differential equations feature enhancement module to augment and amplify the patient representation. A logistic regression was then applied to produce the patient-level prediction, achieving an AUC of 0.768.

The final two patient-level approaches are from the same research group, and both did not use expert LN annotations from the cancer patient set.^16 23^ In a multidomain, multitask approach, Glaser *et al*^23^ used a public dataset with 595 annotated LN locations on CT scans of patients who do not have colon cancer and a private dataset with CT scans of 123 patients with colon cancer. The architecture was a DenseNet encoder-decoder structure which was first trained on the public data to detect LNs and then the encoder was fine-tuned for LNM classification. The idea is that this approach will help refine the classifier, as the encoder generates feature representations that capture information relevant for both classification and for identifying LNs in the image. This approach achieved an AUC of 0.86. Following this study, Bedrikovetski *et al*^16^ used a dataset of 1201 CT scans from patients with colon cancer and segmentations on 500 scans by a non-expert team including a postgraduate researcher, a medical officer and a colorectal surgeon. They followed a similar approach to Glaser *et al*,^23^ training a ResNet-50 segmentation model and then adapting the segmentation encoder to the classification task. The segmentation model was applied to select 40 slices for each patient to be used in the classification MLP model which achieved an AUC of 0.542. They noted that their preliminary study achieved a much higher performance due to its smaller sample size and differences in model architecture.^23^ The study did not provide metrics for the segmentation model, however, the segmentation was fundamental to the classification approach and would help provide context to the results. Given that there were not any annotations for 700 CT scans including all the test cases, it could be that the classification model is performing poorly as a result of not capturing enough of the LNs within the selected 40 slices.

#### Individual LN assessment

Due to the difficulties associated with matching up the pathological status of dissected LNs with the pre-operative radiologic imaging, limited work exists that uses the pathology of individual nodes. Most of the individual LN prediction studies used a subjective ground truth with a less accurate radiologist assessment of LNs. Only two studies employed a collaboration between radiologists and pathologists to match the images to the pathology.^24 25^ The first, Li *et al*^24^ had annotations of LNs from rectal cancer patient MRI scans. They selected the largest node from the mesorectal or rectal superior arterial regions, in total 99 positive and 128 negative LNs from 129 patients. The architecture was an Inception-v3 model trained on ImageNet to transfer feature detection ability to this task with a logistic regression layer for classification. This approach had an AUC of 0.994 and an accuracy of 95.7% and was strengthened by comparing the model directly with radiologists. However, the test set contained just 26 patients so the results may not reflect the true performance. Also, the dataset was split into training and test based on individual nodes rather than at the patient-level. This test set leakage could cause some confounding bias as patients have a unique cancer biology and so the model may be recognising this independently of generalised features. Finally, the task is made significantly easier as they only selected the largest LNs and large malignant LNs often grow into a more spherical shape with a heterogeneous border due to uncontrolled cell division, whereas large benign nodes retain a more typical oval shape.^26 27^ Li *et al*^24^ are from the same research group that produced three other papers included in this review.^28^,^30^

More recently, Ozaki *et al*^25^ used patches of lateral LNs on MRI for rectal cancer patients with or without neo-adjuvant chemoradiotherapy (CT/RT) treatment. Images were split into two groups: baseline (pretreatment) and presurgery (after CT/RT treatment). The straight-to-surgery group had the same scan for both groups. In total, 209 patients were included across 15 institutions of which 124 went straight to surgery, with 52 receiving CT and 33 receiving both CT and RT. Across the patient set, 3547 lateral LNs were dissected and just 97 were pathologically confirmed as positive LNs. They linked the radiology with the pathology using the LN diameters. The model was a ResNet-18 model with transfer learning and a logistic regression layer to classify the individual nodes. The baseline precision-recall AUC (PR-AUC) was 0.870 and the presurgery PR-AUC was 0.963. However, the validation results were given instead of the test results and the sensitivity and specificity were not reported even though the dataset was highly imbalanced with 96.5% negative LNs. Additionally, the same pathology label was used for both the baseline and presurgery images; however, specific nodes could have responded to CT/RT and be malignant on the baseline and benign on the presurgery and pathology.

Five studies are more directly comparable since they all used radiologist assessment of LNM as the ground truth for the models instead of pathology.^28^,^32^ However, this means that the true performance is unlikely to match the reported metrics as radiologic evaluation of rectal cancer LNs on MRI has limited diagnostic sensitivity of 73% and specificity of 74%.^4^ Additionally, there may be a greater variety in the quality of radiologist assessment compared with pathology. This is because the diagnosis is more subjective and will depend on the level of experience and whether an agreement between multiple radiologists was used for staging. The best-performing approach by Lu *et al*^31^ used a faster region-based CNN (Faster R-CNN) architecture with a backbone VGG16 model pretrained on ImageNet for bounding box detection and an MLP for classification. The dataset held 351 rectal cancer MRI scans, annotated by radiologists, including T1, T2 and diffusion weighted. This approach was tested on 414 patients across 6 different medical centres, achieving an AUC of 0.912. All patients had at least one metastatic LN which is not representative of the true incidence rate. Lu *et al*^31^ are from the same research group as two other included studies.^17 32^ Ding *et al*^32^ further evaluated this model with the same test set using correlation and consistency analysis to compare Faster R-CNN with a team of radiologists interpreting the MRI scans, and a team of pathologists using postoperative histology as the ground truth. They also included N-stage-specific comparisons and survival analysis for an enhanced model evaluation. The study found that the deep learning method surpassed the radiologists and had a closer correlation and consistency with pathologists, compared with radiologists with pathologists.

Lastly, three studies from the same research group investigated CRC LNM on MRI.^28^,^30^ In the initial study, Li *et al*^28^ used the Inception-v3 model and an MLP classifier, which had a performance accuracy of 94.4% on a dataset including 312 benign and 307 malignant LNs. The dataset was then expanded to 3364 patients, of which 1646 were positive and 1718 were negative. First, multiple approaches were investigated by Li *et al*,^29^ comparing radiomics with deep learning feature representations using eight machine learning methods, and three approaches which pair an MLP with a large CNN model, either LeNet or AlexNet with and without transfer learning from training on ImageNet. The best-performing model was AlexNet with transfer learning, achieving 0.794 AUC and 75.8% accuracy. The selected model was extended by Li *et al,*^30^ employing skip connections between the convolutional layers to better transfer and combine low-level and high-level features. The resulting model achieved a performance of 0.857 AUC and 83.6% accuracy, and it was further validated by comparing with radiologists.

### Common limitations of the methodology and validation of deep learning approaches

Overall, the existing research has several common areas for improvement regarding the research data, algorithm development and model validation. First, most studies did not include important clinical data or imaging of the primary tumour in their models, even when patients across multiple T-stages were involved. The T-stage describes the extent of the tumour and how far through the bowel wall the cancer has grown. A higher T-stage is more likely to be associated with LNM due to increased access to lymphatic networks.^2^ Only three studies included an image of the primary tumour.^16 21 22^ Additionally, one other study did not have this limitation as they focused on T1-2 stage cancer and included features of the primary tumour.^18^ Only two studies included clinical data alongside imaging data to predict LNM.^17 21^ This is fundamentally different from clinical practice where a radiologist can consider biomarkers such as age as well as related disease-specific characteristics including extramural vascular invasion. Additionally, some studies (n=6) did not provide a clinical summary of the data, therefore, we cannot judge whether the cohorts involved in these studies were fair and representative of the target population. Only a handful of studies used data augmentation^18 22 24 25 28^ and hyperparameter training^17 18 24 29 30^ which are known to improve the performance of deep learning models.

Several of the studies may have limited generalisability due to selection bias, including differences in the proportion of malignant LNs. Models may not perform well in settings with significantly different patient characteristics. The studies usually involved patients who underwent surgery without neo-adjuvant treatment, and recent statistics showed that 38.4% of patients with rectal cancer from this group had LNM in England in 2019.^33^ However, some of the patient-level studies had a proportion of LNM patients which is significantly different from this, including Ding *et al*^17^ (77.3%), Wan *et al*^18^ (22%) and Liu *et al*^21^ (61.4%). There may also be selection bias in the individual LN assessment studies, as there can be significant variance in the LN yields from a given surgery due to factors such as time spent looking for LNs and evolving surgical strategies.^34^ For example, Li *et al*^24^ only selected approximately 1.8 nodes per patient which is much lower than the recommended yield of at least 12 for pathological examination.^34^ Additionally, Li *et al*^29 30^ used a dataset which had approximately 49% positive nodes, however, the true proportion would be much lower in most cases if smaller LNs were included.

Expert manual segmentation was required for every study except for Glaser *et al*^23^ who used transfer learning from a publicly available dataset with expert annotations. Although two studies had a lower difficulty segmentation, Wan *et al*^18^ used patches containing the primary tumour, and Bedrikovetski *et al*^16^ used non-expert annotations which are more likely to be error prone. Expert annotations are expensive and time-consuming to produce, which limits the end-to-end applicability of the existing research as the models cannot be applied without human input to first localise the LNs before diagnosis. Finally, test set size is also a common limiting factor, as all studies except Bedrikovetski *et al*^16^ and Ding *et al*^17^ had less than 100 patients in the testing sets used to generate results.

In general, the standard of validation was low as many studies avoided any validation techniques beyond reporting performance metrics. Advanced model validation techniques were rarely used, Xie *et al’*s study^22^ was the only one included in this review that employed cross-validation, and none used bootstapping. Several studies (n=5) only reported a single metric which can obscure the weaknesses of these models and make it difficult to compare results across studies. Although most studies did include a receiver operating characteristic (ROC) curve to aid model interpretation (n=10), only two studies included a calibration plot.^17 21^ Only a limited number of studies used statistical tests to compare models (n=5). None of the studies did an external validation which would give a better indication of the model generalisability, although five studies did combine training data from multiple institutions.^16 17 25 31 32^

### Explainability and interpretability of deep learning methods

While most deep learning research on this task is inherently not explainable or interpretable, some studies had components which help to improve this aspect. First, two studies included clinical data in their nomogram models.^17 21^ This approach incorporates multimodal data as a clinician would, and it explicitly shows the contribution of each factor to the diagnosis which may help to interpret the results. Three studies used Grad-CAM heatmaps as a post-hoc explainability technique to indicate the areas that were highly important to the diagnosis.^18 24 30^ First, two studies provided the same example with the area containing the LN highlighted by Grad-CAM.^24 30^ Additionally, the example in Wan *et al*^18^ suggested that areas both inside the primary tumour and close to it were important for the diagnosis. While this provides some evidence that the models were focused on clinically important regions, limited examples were given, and they would have been improved by including hard-to-classify cases.

Attention mechanisms can allow a model to focus on the important areas while discarding other information, this is analogous to human vision which provides some inherent explainability. Also, attention mechanisms can be used to inspect a prediction by ordering the importance of the components such as the slices, patches or individual LNs. Xie *et al’*s study^22^ is the only one to use an attention mechanism, the MIL bag of instances representation combined both local LN and global slice information, providing a habitat imaging approach which takes into account the local surroundings of the LNs as a clinician would. Although, they did not use the attention scores to enhance model interpretability.

## Discussion

This scoping review provides an overview of the existing research on deep learning methods applied to CRC LNM prediction using pre-operative radiologic imaging. The AUC scores with a 95% CI for the deep learning methods were found to be 0.856 (0.796 to 0.916) for patient-level models (not including the outlier result from Bedrikovetski *et al*^16^) and 0.904 (0.841 to 0.967) for diagnosis of specific LNs.

In answer to the first research question on limitations, the existing research has limited generalisability as many studies are held back by fundamental issues with the data, methodology and validation. Several studies have focused exclusively on the radiologic appearance of LNs, however, clinicians use multimodal data about the patient to inform these diagnoses. Incorporating primary tumour information would help as there are different proportions of LNM across T-stages. Most studies that did individual LN prediction had invalid results due to using less accurate radiologic staging instead of pathology as the ground truth. Comparisons between studies are challenging as they all rely on private datasets. Some of these datasets had selection bias due to differences in the prevalence of LNM, and several did not report a clinical summary of the patient set. Other flaws in the methodology include reporting limited metrics, small testing sets, limited hyperparameter training and not using data augmentation. Finally, only one study used cross-validation and zero did an external validation.

On the second research question related to the explainability of the approaches, most of the existing deep learning research on this task is fundamentally not explainable or interpretable. Only three studies attempted to explain the black-box models, using Grad-CAM gradient heatmaps to show that clinically relevant regions were important in the classification.^18 24 30^ Two studies included clinical data in a nomogram to provide a more interpretable diagnosis, which weights the contribution of the deep learning model prediction and other biomarkers. Additionally, Xie *et al*^22^ used an attention mechanism and combined images of LNs with a wider context from whole slices, this is arguably towards more explainable models as the attention mechanism explicitly weights the contribution of each LN to a patient-level diagnosis, and so they can indicate the most suspicious LNs.

In comparison to radiomics research, Ma *et al*^35^ provide an excellent example of robust modelling and validation. The dataset, provided with a comprehensive clinical summary, held a total of 519 rectal cancer patient MRI scans including an external validation cohort from a different hospital. They used the self-configuring segmentation model nnUNet^36^ for automatic localisation of the primary tumour, which achieved a mean Dice score of 0.857. The patient-level LNM model was a clinical-radiomic nomogram. Radiomic feature selection was performed using both the SelectKBest and the least absolute shrinkage and selection operator (LASSO) algorithms. Clinical data were included in the model based on Pearson’s χ^2^ test and Fisher’s exact test for categorical variables, as well as Student’s t-test and Mann-Whitney U test for continuous data. The validation techniques included using multiple metrics, ROC curves and cross-validation. Additionally, they assessed the goodness-of-fit of the nomogram using the Hosmer-Lemeshow test and calibration curves which indicated an ideal fit. They used decision curve analysis against a combined diagnosis from three experienced radiologists and the model using only radiomics evaluation (Radscore) without clinical data, concluding that the clinical-radomic nomogram was a net benefit over both. The final model had a higher AUC than the Radscore and radiologists in both the internal validation cohort using a temporal split in patients (0.908 vs 0.735 vs 0.640) and the external validation cohort (0.884 vs 0.802 vs 0.734), and these were confirmed as statistically significant improvements using the DeLong test. This study demonstrates established validation practices and contrasts with the lack of thorough model validation within the deep learning-based research for CRC LNM prediction.

This literature review has a significantly different and more focused scope than the existing reviews.^9 10^ This review also captures recent developments in the field including five studies from 2023 which were not included in the previous reviews. Additionally, it follows the rigorous reporting guidelines of the PRISMA-ScR protocol,^15^ strengthening the consistency across studies, and the transparency and completeness of the review. However, the search, study selection and data extraction were conducted by one person (BK) which is a limitation as the findings may be subject to bias or oversight, although extensive note-taking and checks were performed to mitigate these risks. The research questions have a comparatively more focused scope than the other LNM staging review articles as we provide an in-depth analysis of the deep learning research and look at more granular comparisons. Although this is less informative of the wider picture, it allows a more technical-oriented overview of the knowledge base to inform further research using deep learning.

Now, reflecting on the findings of this review, we provide several important insights. First, comparisons across studies are challenging due to the inconsistency of the data and therefore the field would be helped by a benchmark dataset with a representative sample across multiple hospitals with expert annotations. Second, the studies based on individual LN assessment were fundamentally limited by using radiologist assessment or only matching with the pathology for the largest nodes by using the diameter. However, a patient-level approach which includes all LNs and aims to indicate the most suspicious ones may be more robust and also require less expert input to curate the dataset. Third, while four studies used CT, MRI is the recognised gold standard for imaging CRC due to its higher level of detail. However, CT offers more uniformity and consistency across different scanners.^3^ As a result, multimodal approaches may be preferable to leverage both of their strengths, though MRI would be recommended for single-modality studies. Next, most of the studies used large and pretrained CNNs, sometimes with modifications, indicating this is the current state-of-the-art approach. However, the existing research is not using the latest advances in generative artificial intelligence (GenAI), vision transformers and attention mechanisms which may help improve the performance and interpretability. Very few studies used automatic segmentation or other methods to localise the LNs, which would make the models end-to-end and more transferable to clinical practice. None of the included studies did an analysis of hard-to-classify cases such as small malignant nodes or analysed incorrect predictions in an attempt to find the areas where the model is performing better or worse. Further research would benefit from highlighting uncertain predictions to recommend for expert review. Finally, we suggest that future research could apply the increasingly popular explainable artificial intelligence (XAI) technique of concept bottleneck models.^37^ The model explicitly makes individual predictions for clinically relevant features in an intermediate layer before the final diagnosis, this is inherently more explainable as it allows clinicians to inspect and update the model predictions at the feature level.

In summary, existing research indicates that it is possible to achieve superhuman performance, exceeding the sensitivity and specificity of 73% and 74% of radiologists.^4^ Within CRC LNM prediction, several methodological and validation flaws make it difficult to trust the results and current approaches lack explainability to help clinical interpretation. This presents a research gap for deep learning models that are end-to-end with automatic localisation of the LNs, are representative of the target population and are robustly validated including external validation and comparisons with radiologists. Furthermore, additional efforts are needed to enhance model interpretability and to improve clinical trust.

## supplementary material

10.1136/bmjopen-2024-086896 online supplemental file 1

10.1136/bmjopen-2024-086896 online supplemental file 2

## Data Availability

No data are available.
