# Supplementary material for: State-of-the-art performance of deep learning methods for pre-operative radiologic staging of colorectal cancer lymph node metastasis: a scoping review
Source: BMJ Open. 2024 Dec 2;14(12):e086896. doi: 10.1136/bmjopen-2024-086896 (PMC11624802; doi:10.1136/bmjopen-2024-086896)
Supplement: online supplemental file 1 [file bmjopen-14-12-s001.docx]

**Supplementary Material 1**

**Search Terms:**

The academic databases were searched on the 1^st^ February 2024 using the following search terms and restrictions.

**Embase/MEDLINE:**

Produced 54 results

| 1. | rectal |
| --- | --- |
| 2. | colorectal |
| 3. | cancer |
| 4. | ai |
| 5. | machine learning |
| 6. | artificial intelligence |
| 7. | deep learning |
| 8. | lymph node |
| 9. | mr |
| 10. | mri |
| 11. | ct |
| 12. | 1 or 2 |
| 13. | 8 or 9 |
| 14. | 4 or 5 or 6 or 7 |
| 15. | radiology |
| 16. | 10 or 11 or 12 or 16 |
| 17. | 3 and 13 and 14 and 15 and 17 |
| 18. | limit 18 to yr="2018 -Current" |
| 19. | limit 19 to english language |
| 20. | limit 20 to full text |
| 21. | remove duplicates from 21 |

**Scopus:**

( TITLE-ABS-KEY ( "rectal" ) OR TITLE-ABS-KEY ( "colorectal" ) ) AND TITLE-ABS-KEY ( "cancer" ) AND ( TITLE-ABS-KEY ( "artificial intelligence" ) OR TITLE-ABS-KEY ( "machine learning" ) OR TITLE-ABS-KEY ( "deep learning" ) ) AND ( TITLE-ABS-KEY ( "lymph node" ) AND ( TITLE-ABS-KEY ( "mr" ) OR TITLE-ABS-KEY ( "mri" ) OR TITLE-ABS-KEY ( "ct" ) OR TITLE-ABS-KEY ( "radiology" ) )

Restrictions: “2018-2024” and “Document type: Article or Review or Conference paper”

Produced 46 results

**IEE Xplore**

("Full Text Only": rectal OR "Full Text Only": colorectal) AND ("Full Text Only": cancer) AND ("Full Text Only": artificial intelligence OR "Full Text Only": machine learning OR "Full Text Only": deep learning) AND ("Full Text Only": lymph node) AND ("Full Text Only": mr OR "Full Text Only": mri OR "Full Text Only": ct OR "Full Text Only": radiology)

Restrictions: “2018-2024” and “Filters: Journals or Conferences”

Produced 82 results

**Web of science:**

(ALL=(rectal) OR ALL=(colorectal)) AND ALL=(cancer) AND (ALL=(artificial intelligence) OR ALL=(machine learning) OR ALL=(deep learning)) AND ALL=(lymph node) AND (ALL=(mr) OR ALL=(mri) OR ALL=(ct) OR ALL=(radiology))

Restrictions: “2018-2024”

Produced 55 results

**Google Scholar:**

(“colorectal” or “colon” or “rectal”) and (“cancer”) and (“ai” or (“artificial” and “intelligence”) or (“deep” and “learning”) or (“machine” and “learning”)) and (“lymph” and “node”) and (“mr” or “mri” or “ct” or “radiology”)).

Restrictions: “2018-2024”

Note that Google Scholar was searched in a non-systematic way, the reviewer BGK selected studies to read the abstract based on the title whereas the abstracts were reviewed for all results on the other databases.
