# Supplementary material for: State-of-the-art performance of deep learning methods for pre-operative radiologic staging of colorectal cancer lymph node metastasis: a scoping review
Source: BMJ Open. 2024 Dec 2;14(12):e086896. doi: 10.1136/bmjopen-2024-086896 (PMC11624802; doi:10.1136/bmjopen-2024-086896)
Supplement: online supplemental file 2 [file bmjopen-14-12-s002.docx]

**Supplementary Material 2**

**Protocol for scoping review:**State-of-the-art performance of deep learning methods for pre-operative staging of colorectal cancer lymph node metastasis: a scoping review

**Abstract:**

**Introduction:** This scoping review will assess the current state-of-the-art in deep learning methods applied to pre-operative colorectal cancer lymph node metastasis prediction. Specifically, evaluating the data, methodology, and validation of existing work and the current use of explainable AI in this domain.
**Methods and analysis:** The methodology for the review will involve searching the academic databases MEDLINE, Embase, Scopus, IEEE and Web of Science with defined terms, screening by title and abstract, choosing a subset to assess the full text, and including the articles which meet the inclusion criteria. This scoping review and the reporting of findings will be guided by the PRISMA-ScR protocol.
**Ethics and dissemination:** The scoping review will not require ethics approval. It is being conducted as part of a PhD project and will be handed in as part of the doctoral thesis and submitted to a peer-reviewed journal.

**Introduction:**

In colorectal cancers, lymph node metastasis is an important clinical feature which influences treatment. If cancer spreads to the lymph nodes it can lead to further metastasis to other parts of the body. Therefore when it is identified it is often recommended to have neo-adjuvant chemoradiotherapy to downstage the lymph nodes before surgery. However, clinical rules-based criteria for the assessment of lymph nodes currently have sensitivity and specificity of 73% (95% CI, 68-77%) and 74% (95% CI, 68-80%) respectively [1]. Inaccurate lymph node staging can lead to inappropriate treatment and in some cases a worsened prognosis for the patient.

In recent years, radiomics features with machine learning methods have been applied to this clinical task and have become well established with robust internal and external validation. More recently, deep learning methods have been applied and a recent systematic review by Bedrikovetski et al. [2] found that for rectal cancer, there was a per patient AUC of 0.917 (0.882–0.952) for deep learning models, 0.808 (0.739–0.876) for radiomics, and 0.688 (0.603-0.772) for radiologists. Indicating that both machine learning approaches were better than radiologists at this important clinical task, and that deep learning image features and methods have a higher performance potential than radiomics based approaches. This scoping review aims to bridge the gap between the systematic review to the present where deep learning approaches have advanced considerably since then and there is an increasing frequency of publications in this domain. This review aims to provide an in-depth showcase of the current knowledge base for deep learning methods for colorectal cancer lymph node metastasis.

**Research questions:**

1. What is the current state of the art for deep learning based methods applied to predict lymph node metastasis in colorectal cancer using pre-operative radiological imaging?

2. What are the common pitfalls and limitations of the methodology and validation of the deep learning approaches?

3. To what extent are the existing deep learning based methods explainable and interpretable?

**Objectives:**

This scoping review will assess the current state of the art in deep learning methods applied to pre-operative colorectal cancer lymph node metastasis prediction. We will evaluate each paper identified based on the data, methodology and validation, aiming to identify any limitations which devalue their reported performance metrics. Additionally, we will look for evidence of the use of explainable AI in this domain.

**Methods and analysis:**

This scoping review and the reporting of its results will be guided by the Preferred Reporting Systems for Systematic Reviews and Meta-Analyses Protocols-Extension for Scoping Reviews (PRISMA-ScR) protocol [3]. The academic databases Embase, Scopus, IEEE, and Web of Science will be searched with defined terms, results will be screened by the title and abstract to identify papers to be assessed by the full text. Additionally, we will look for papers on Google Scholar and perform snowball searching by reviewing the reference list of each of the included studies. Full text articles will be carefully read and checked to ensure all the inclusion criteria are met and studies will be selected for inclusion. Next, an extraction template will be used to compile information from each of the included studies. The template is used to record the results, to ensure the same information is collected from each study, and to allow better comparisons.

**Search terms:**

The following search terms will be used to identify relevant papers from the full text:
(“colorectal” or “colon” or “rectal”) and (“cancer”) and (“ai” or (“artificial” and “intelligence”) or (“deep” and “learning”) or (“machine” and “learning”)) and (“lymph” and “node”) and (“mr” or “mri” or “ct” or “radiology”)).

**Inclusion and exclusion criteria:**

The scoping review will include any studies published since 2018 which apply deep learning methods for both the feature extraction and classification of colorectal cancer lymph nodes on preoperative radiological imaging. The defined inclusion and exclusion criteria are given in Table 1.

**Table 1:** Inclusion and exclusion criteria for the literature review.


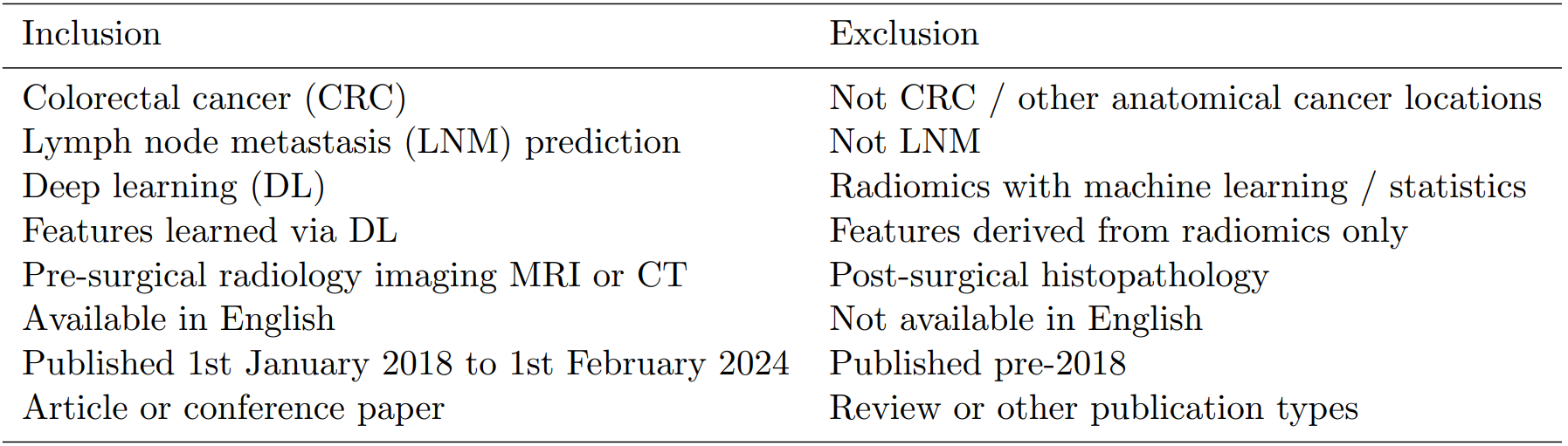


**Extraction template:**

The following extraction template, which will be converted to an Excel spreadsheet, will be used to collect the same information from each study and to keep a record of the results.

| Study Details (title, 1^st^ author, year of publication, document type, web link, code availability) |  |
| --- | --- |
| Inclusion and exclusion criteria |  |
| Cancer location (colorectal, colon, rectal) |  |
| Number of participants and number of lymph nodes with breakdown of positive/negative status (train, validation, test) |  |
| Data type (CT, MRI, clinical data, 2D or 3D, patient or node level classification) |  |
| Metrics available (train/test AUC, accuracy, sensitivity, specificity etc.) |  |
| Methods used (e.g. CNN, features/variables, attention, segmentation) |  |
| Limitations of methodology (incorrect or inappropriate methods, test set leakage etc.) |  |
| Validation techniques used (metrics, ROC curves, , statistical tests, cross validation, external validation etc.) |  |
| Limitations of validation |  |
| Explainability and interpretability of approach (e.g. Grad-CAM, attention, clinical information) |  |
| Comments / anything relevant to my future work |  |
| Snowball literature search findings |  |

**References:**

1. Zhuang Z, Zhang Y, Wei M, Yang X, Wang Z. Magnetic Resonance Imaging Evaluation of the Accuracy of Various Lymph Node Staging Criteria in Rectal Cancer: A Systematic Review and Meta-Analysis. Front Oncol. 2021;**11**(July).
   <https://doi.org/10.3389/fonc.2021.709070>
2. Bedrikovetski S, Dudi-Venkata NN, Kroon HM, et al. Artificial intelligence for pre-operative lymph node staging in colorectal cancer: a systematic review and meta-analysis. BMC Cancer. 2021;**21**(1):1058.
   <https://doi.org/10.1186/s12885-021-08773-w>
3. Tricco AC, Lillie E, Zarin W, et al. PRISMA extension for scoping reviews (PRISMA-ScR): Checklist and explanation. Ann Intern Med. 2018;**169**(7):467–73.
   <https://doi.org/10.7326/M18-0850>
