# Supplementary material for: State-of-the-art performance of deep learning methods for pre-operative radiologic staging of colorectal cancer lymph node metastasis: a scoping review
Source: BMJ Open. 2024 Dec 2;14(12):e086896. doi: 10.1136/bmjopen-2024-086896 (PMC11624802; doi:10.1136/bmjopen-2024-086896)
Supplement: Supplementary file 3 [file bmjopen-2024-086896.reviewer_comments.pdf]

PEER REVIEW HISTORY

BMJ Open publishes all reviews undertaken for accepted manuscripts. Reviewers are asked to complete a checklist review form and are provided with free text boxes to elaborate on their assessment. These free text comments are reproduced below.

ARTICLE DETAILS

Title (Provisional)

State-of-the-art performance of deep learning methods for pre-operative radiologic staging of colorectal cancer lymph node metastasis: a scoping review

Authors

Keel, Benjamin George; Quyn, Aaron; Jayne, David; Relton, Samuel David

---

VERSION 1 - REVIEW

**Reviewer**	**1**
**Name**	**Schüffler , Peter**
**Affiliation**	**Technical University of Munich**
**Date**	**19-Apr-2024**
**COI**	**none**

The authors review current AI models for lymph node detection in pre-operative CRC.

They acknowledge previous reviews in the field from 2022 and aim to extend them with more recent publications.

The review is well-structured, nicely written, and certainly helpful in the field.

Still, I recommend revision to address some critical points:

- Please discuss the data sets used in the studies for training and testing. Are these public data sets? All proprietary? Research fellows will benefit from this information, especially if these are public data sets.

- You claim that you can go a bit more in-depth in this review. I haven't read so much in that direction... For example, can you state which models/architectures are the most successful ones? On P21, you already list which studies use which architectures, but to gain knowledge of it, a relation to their success rate would be required. What can we learn from all these studies?

- Are the presented models generalizable? Do they use inhouse/outhouse validation?

- How well are the validation/test AUCs and accuracies comparable to each other? Do we need benchmark data sets?

- Based on the multiple studies, can you state that LN detection, in principle, works better on CT or on MRI or equally well? Would a multimodal approach using CT and MRI make sense?

- P27, L24: Dice with a capital D (it is a name)

- P28, L52: superhuman performance? what is that, who indicates that? Please cite.

**Reviewer**	**2**
**Name**	**Chong, Yosep**
**Affiliation**	**The Catholic University of Korea, Hospital Pathology**
**Date**	**01-May-2024**
**COI**	**none**

This is a relatively well organized review paper with interesting topic. However there are some parts that need improvement.

1. Title : It is better to mention clearly that this is about "radiologic" pre-operative staging AI.

2. Abstract : Please include why we need this review in the objective. It seems to be rather lengthy and verbose. Please include suggestion for the future study design in the conclusion and reduce the overall length by removing redundant sentences.

3. Introduction:

It is generally well written but too lengthy. Please make it slightly brief than now like reducing to 3 pages.

4. Methods:

5. Results: Table 2 should be the key finding of this study but it is way too simple. Please add countries where each study was conducted (or ethnicity of the target population), Please add baseline AI model, Please rearrange the table according to the data type (CT or MRI), please add detailed information about the data such as stage or inclusion of chemotherapy, task type (classification, segmentation, etc.)

Please make figure that effectively present the accuracy or AUC of all the included studies within a figure.

There are too few figures or tables. It is basically very descriptive summarizing each study design and findings. It is too lengthy and hard to read. Please categorize similar studies into a certain group and mention only few studies that are mentionable. Use figures to present types of study design (individual LN detection, box-level detection, patient-level prediction) and present a few exemplary figures from each study that can effectively explain study overview and AI model design to effectively help readers to understand each study design method better.

Unify the numbers of digits below zero within tables or manuscript.

I guess this journal does not allow using citations in the middle of sentence or as words. Please refer author's instruction once again and correct citations accordingly.

Results are too lengthy. Please reduce the length. (13.5 pages -> 7-8 pages)

Some of the part in results can be moved to discussion part. Discussion is relatively short. Authors pointed out common limitations of previous studies but did not properly suggest what should be included or concerned for better study design. Appropriate number of the data, appropriate way of annotation and setting up ground truth in different settings. Authors mentioned one good study but it is not enough. Authors can do better by suggesting better study design for future studies.

There are some awkward expression or incomplete sentences throughout the manuscript. please check manuscript carefully and revise English once again.

Please cite these references if appropriate in the relevant area.

DOI: 10.3390/cancers14112590

DOI: 10.1093/bib/bbad151

DOI: 10.3390/cancers12071884

VERSION 1 - AUTHOR RESPONSE

Review 1

Thank you Prof. Peter Schüffler for the positive feedback and the insightful comments and suggestions.

1. *Please discuss the data sets used in the studies for training and testing. Are these public data sets? All proprietary? Research fellows will benefit from this information, especially if these are public data sets.*

Response:

All the datasets are private and this is now mentioned in the Discussion. There is a summary of the datasets for each study in Table 2 and the “Current state-of-the-art” section and we have expanded the discussions and comparisons between datasets in the “Common limitations” section and the 2nd and 6th (2nd last) paragraphs in the Discussion. These discussions include reporting of clinical summaries of the data, whether the data includes patients from multiple institutions, use of imaging of the primary tumour or T-stage, use of patients across multiple T-stages and the proportion of lymph node metastasis.

2. *You claim that you can go a bit more in-depth in this review. I haven't read so much in that direction... For example, can you state which models/architectures are the most successful ones? On P21, you already list which studies use which architectures, but to gain knowledge of it, a relation to their success rate would be required. What can we learn from all these studies?*

Response:

We have included a summary paragraph on the architectures and baseline comparisons at the start of “Current state-of-the-art” and the 2nd last paragraph in the Discussion including the current architectures being used and suggesting other techniques. Additionally, we added a summary of the architectures in the expanded Table 2 to provide readers with an opportunity to compare architectures and weigh this in the context of their results and

limitations. Figure 3 has been included to present the optimal study design based on the best components and architecture of existing studies. Finally, the Discussion was expanded to go more in-depth and to give some insights from the review.

3. *Are the presented models generalizable? Do they use inhouse/outhouse validation?*

Response:

It is better to frame the discussions of studies in terms of generalisability as this infers their potential for clinical use. We have updated the text to make the link between study design and generalisability clearer. Also, all of the included studies only use inhouse validation without external/outhouse validation.

Both of these topics are covered in the "Common limitations" section of the Results and the summary of this section in the Discussion (2nd paragraph).

4. *How well are the validation/test AUCs and accuracies comparable to each other? Do we need benchmark data sets?*

Response:

We have added Figure 2 to compare studies based on model performance metrics. However, each study has separate and private data with different malignant proportions and some studies have fundamental limitations in the methodology and validation making comparisons difficult. So yes, a benchmark dataset would be of great help to the field and this is an excellent point now included in the Discussion.

5. *Based on the multiple studies, can you state that LN detection, in principle, works better on CT or on MRI or equally well? Would a multimodal approach using CT and MRI make sense?*

Response:

This is another good discussion point. In principle, MRI provides higher detail than CT although MRI can be less generalisable due to differences between scanners which are especially pronounced across hospitals. Whereas, the appearance of CT scans is more uniform. A multimodal approach may have some advantages in picking up details unique to one of the modalities. We have added a couple of sentences on this to the Discussion.

6. *P27, L24: Dice with a capital D (it is a name)*

Response:

This has been updated thanks for noticing it.

7. *P28, L52: superhuman performance? what is that, who indicates that? Please cite.*

Response:

This claim is uncited, thanks for pointing it out. Superhuman refers to the models having a higher diagnostic performance than human radiologists. The reference for this is the 73% sensitivity and 74% specificity computed in a systematic review of (human) MRI evaluation of lymph node staging from 2021. This statement now has the following citation added (https://doi.org/10.3390/jcm11092599)

Review 2

Thank you Dr. Yosep Chong for the detailed comments and suggestions for improving the readability and usability of this review.

We have updated the numbering of each comment to organise our response.

1. *Title: It is better to mention clearly that this is about "radiologic" pre-operative staging AI.*

Response:

Thanks for the suggestion, we have changed the title to this.

2. *Abstract: Please include why we need this review in the objective. It seems to be rather lengthy and verbose. Please include suggestion for the future study design in the conclusion and reduce the overall length by removing redundant sentences*.

Response:

The abstract has been edited to be more concise. We have indicated we need the review as this is a fast moving domain. In the Conclusion, we have summarised the key methodological flaws in existing study designs.

3. *Introduction: It is generally well written but too lengthy. Please make it slightly brief than now like reducing to 3 pages.*

Response:

Some general editing has been done to shorten where possible to remove repeated information and to improve the flow, the introduction is now just over 3 pages and the word count is reduced by 150.

4. *Results: Table 2 should be the key finding of this study but it is way too simple. Please add countries where each study was conducted (or ethnicity of the target population), Please add baseline AI model, Please rearrange the table according to the data type (CT or MRI), please add detailed information about the data such as stage or inclusion of chemotherapy, task type (classification, segmentation, etc.)*

Response:

Thank you for these suggestions, we have expanded Table 2 to be more useful to the reader by including further columns with the year, country, model architecture/task and a key limitation to allow more detailed comparisons with added context. For the T stage, all studies except for Wan et al. [17] had patients across all T stages which has now been noted as a unique aspect of [17] in the table and the text. Additionally, Ozaki et al. [21] was the only study to include chemo-radiotherapy patients and this is indicated as a unique aspect. Finally, we have included which studies compared to baseline models or radiologists in the first paragraph under “Current state-of-the-art”.

5. *Please make figure that effectively present the accuracy or AUC of all the included studies within a figure.*

Response:

We have included Figure 2 to present and compare the metrics across the included studies.

6. *There are too few figures or tables. It is basically very descriptive summarizing each study design and findings. It is too lengthy and hard to read. Please categorize similar studies into a certain group and mention only few studies that are mentionable. Use figures to present types of study design (individual LN detection, box-level detection, patient-level prediction) and present a few exemplary figures from each study that can effectively explain study overview and AI model design to effectively help readers to understand each study design method better.*

Response:

We acknowledge that in particular the “Current state-of-the-art” section of the Results was quite lengthy with too much description in places. We have been through this section carefully to remove unnecessary information where there is too much description of a specific study.

The included work has been grouped as much as possible, some studies are discussed together when they are from the same research group, and studies are grouped when there are similarities such as imaging modality (MRI or CT) and if it is patient level or individual LN assessment. The structure of the “Current state-of-the-art” section is to go through each separate study in detail and since there are a low number of studies we have mentioned all of them explicitly in the text to give a full picture of the existing knowledge base.

We have also included Figure 3 which details a recommended study design based on the best components of the existing approaches. It is included before specific studies are discussed to provide a reference point for readers.

7. *Unify the numbers of digits below zero within tables or manuscript.*

Response:

This has been standardised now.

8. *I guess this journal does not allow using citations in the middle of sentence or as words. Please refer author's instruction once again and correct citations accordingly.*

Response:

We’ve removed all the instances where it uses the citation as a word in the text, it appears from previous articles that it is possible to cite in the middle of a sentence

9. *Results are too lengthy. Please reduce the length. (13.5 pages -> 7-8 pages)*

Response:

We agree the Results was quite lengthy, we have been through it thoroughly to remove sentences which do not add anything and to simplify the phrasing where possible to make it generally less verbose. In total, we have cut around 700 words from the Results and the main text it is now 10.5 pages. We feel that reducing further would impact the completeness of the results.

10. *Some of the part in results can be moved to discussion part. Discussion is relatively short. Authors pointed out common limitations of previous studies but did not properly suggest what should be included or concerned for better study design. Appropriate number of the data, appropriate way of annotation and setting up ground truth in different settings. Authors mentioned one good study but it is not enough. Authors can do better by suggesting better study design for future studies.*

Response:

Thanks for this guidance we have now shortened the Results and expanded the Discussion. We have added a new paragraph (the 2nd last in the Discussion) to provide insights gained from this review and in that we recommend a more robust study design. These include: a discussion of the current architectures being used and suggesting some other techniques, recommending patient level studies, recommending using automatic segmentation methods based on expert annotations to identify lymph nodes, indicating uncertain predictions for human review and improved validation and explainability to increase clinical transferability. However, note

that we have not recommended a dataset size as it has been repeatedly demonstrated that larger datasets are better in computer vision, and it is closely linked to the model complexity.

11. *There are some awkward expression or incomplete sentences throughout the manuscript. please check manuscript carefully and revise English once again.*

Response:

We have been over the manuscript a few times to ensure complete sentences, we corrected grammatical errors and have changed the awkward wording in some areas.

12. *Please cite these references if appropriate in the relevant area.*
    DOI: 10.3390/cancers14112590
    DOI: 10.1093/bib/bbad151
    DOI: 10.3390/cancers12071884

Response:

Thank you for sharing these well structured systematic reviews. We have cited the review on AI for colorectal cancer pathology (https://doi.org/10.3390/cancers12071884) in the introduction to provide evidence for the state-of-the-art performance of AI in colorectal cancer imaging.

VERSION 2 - REVIEW

**Reviewer**	**1**
**Name**	**Schüffler , Peter**
**Affiliation**	**Technical University of Munich**
**Date**	**15-Oct-2024**
**COI**	

I thank the authors for their revision. The authors addressed all my questions.

Final minor remarks are:

- You might want to consider to bring Table 2 into the main text (to me, it appears as a suppl. table).

- You might want to consider a tool for English grammar, some sentences are a bit strange (e.g., "Now, to discuss some insights based on the findings of this review., "Therefore, a patient level approach which includes all LNs and aims to indicate the most suspicious ones may be more robust and also require less expert input to curate the dataset." Therefore -> However?). Everything is understandable, but could be polished a bit.

VERSION 2 - AUTHOR RESPONSE

Now, we will address the final comments from the reviewer: Prof. Peter Schüffler.

1. You might want to consider to bring Table 2 into the main text (to me, it appears as a suppl. table).

Response:

Table 2 has been moved back to the main text.

2. You might want to consider a tool for English grammar, some sentences are a bit strange (e.g., "Now, to discuss some insights based on the findings of this review., "Therefore, a patient level approach which includes all LNs and aims to indicate the most suspicious ones may be more robust and also require less expert input to curate the dataset." Therefore -> However?). Everything is understandable, but could be polished a bit.

Response:

Thanks for these examples, we have corrected them and been through the manuscript carefully to identify and fix any phrasing errors throughout.

<u>Review 1</u>

Thank you Prof. Peter Schüffler for the positive feedback and the insightful comments and suggestions.

8. *Please discuss the data sets used in the studies for training and testing. Are these public data sets? All proprietary? Research fellows will benefit from this information, especially if these are public data sets.*

Response:

All the datasets are private and this is now mentioned in the Discussion. There is a summary of the datasets for each study in Table 2 and the "Current state-of-the-art" section and we have expanded the discussions and comparisons between datasets in the "Common limitations" section and the 2nd and 6th (2nd last) paragraphs in the Discussion. These discussions include reporting of clinical summaries of the data, whether the data includes patients from multiple institutions, use of imaging of the primary tumour or T-stage, use of patients across multiple T-stages and the proportion of lymph node metastasis.

9. *You claim that you can go a bit more in-depth in this review. I haven't read so much in that direction... For example, can you state which models/architectures are the most successful ones? On P21, you already list which studies use which architectures, but to gain knowledge of it, a relation to their success rate would be required. What can we learn from all these studies?*

Response:

We have included a summary paragraph on the architectures and baseline comparisons at the start of "Current state-of-the-art" and the 2nd last paragraph in the Discussion including the current architectures being used and suggesting other techniques. Additionally, we added a summary of the architectures in the expanded Table 2 to provide readers with an opportunity to compare architectures and weigh this in the context of their results and limitations. Figure 3 has been included to present the optimal study design based on the best components and architecture of existing studies. Finally, the Discussion was expanded to go more in-depth and to give some insights from the review.

10. *Are the presented models generalizable? Do they use inhouse/outhouse validation?*

Response:

It is better to frame the discussions of studies in terms of generalisability as this infers their potential for clinical use. We have updated the text to make the link between study design and generalisability clearer. Also, all of the included studies only use inhouse validation without external/outhouse validation.

Both of these topics are covered in the "Common limitations" section of the Results and the summary of this section in the Discussion (2nd paragraph).

11. *How well are the validation/test AUCs and accuracies comparable to each other? Do we need benchmark data sets?*

Response:

We have added Figure 2 to compare studies based on model performance metrics. However, each study has separate and private data with different malignant proportions and some studies have fundamental limitations in the methodology and validation making comparisons difficult. So yes, a benchmark dataset would be of great help to the field and this is an excellent point now included in the Discussion.

12. *Based on the multiple studies, can you state that LN detection, in principle, works better on CT or on MRI or equally well? Would a multimodal approach using CT and MRI make sense?*

Response:

This is another good discussion point. In principle, MRI provides higher detail than CT although MRI can be less generalisable due to differences between scanners which are especially pronounced across hospitals. Whereas, the appearance of CT scans is more uniform. A multimodal approach may have some advantages in picking up details unique to one of the modalities. We have added a couple of sentences on this to the Discussion.

13. *P27, L24: Dice with a capital D (it is a name)*

Response:

This has been updated thanks for noticing it.

14. *P28, L52: superhuman performance? what is that, who indicates that? Please cite.*

Response:

This claim is uncited, thanks for pointing it out. Superhuman refers to the models having a higher diagnostic performance than human radiologists. The reference for this is the 73% sensitivity and 74% specificity computed in a systematic review of (human) MRI evaluation of lymph node staging from 2021. This statement now has the following citation added (https://doi.org/10.3390/jcm11092599)

Review 2

Thank you Dr. Yosep Chong for the detailed comments and suggestions for improving the readability and usability of this review.

We have updated the numbering of each comment to organise our response.

13. *Title: It is better to mention clearly that this is about "radiologic" pre-operative staging AI.*

Response:

Thanks for the suggestion, we have changed the title to this.

14. *Abstract: Please include why we need this review in the objective. It seems to be rather lengthy and verbose. Please include suggestion for the future study design in the conclusion and reduce the overall length by removing redundant sentences*.

Response:

The abstract has been edited to be more concise. We have indicated we need the review as this is a fast moving domain. In the Conclusion, we have summarised the key methodological flaws in existing study designs.

15. *Introduction: It is generally well written but too lengthy. Please make it slightly brief than now like reducing to 3 pages.*

Response:

Some general editing has been done to shorten where possible to remove repeated information and to improve the flow, the introduction is now just over 3 pages and the word count is reduced by 150.

16. *Results: Table 2 should be the key finding of this study but it is way too simple. Please add countries where each study was conducted (or ethnicity of the target population), Please add baseline AI model, Please rearrange the table according to the data type (CT or MRI), please add detailed information about the data such as stage or inclusion of chemotherapy, task type (classification, segmentation, etc.)*

Response:

Thank you for these suggestions, we have expanded Table 2 to be more useful to the reader by including further columns with the year, country, model architecture/task and a key limitation to allow more detailed comparisons with added context. For the T stage, all studies except for Wan et al. [17] had patients across all T stages which has now been noted as a unique aspect of [17] in the table and the text. Additionally, Ozaki et al. [21] was the only study to include chemo-radiotherapy patients and this is indicated as a unique aspect. Finally, we have included which studies compared to baseline models or radiologists in the first paragraph under "Current state-of-the-art".

17. *Please make figure that effectively present the accuracy or AUC of all the included studies within a figure.*

Response:

We have included Figure 2 to present and compare the metrics across the included studies.

18. *There are too few figures or tables. It is basically very descriptive summarizing each study design and findings. It is too lengthy and hard to read. Please categorize similar studies into a certain group and mention only few studies that are mentionable. Use figures to present types of study design (individual LN detection, box-level detection, patient-level prediction) and present a few exemplary figures from each study that can effectively explain study overview and AI model design to effectively help readers to understand each study design method better.*

Response:

We acknowledge that in particular the "Current state-of-the-art" section of the Results was quite lengthy with too much description in places. We have been through this section carefully to remove unnecessary information where there is too much description of a specific study.

The included work has been grouped as much as possible, some studies are discussed together when they are from the same research group, and studies are grouped when there are similarities such as imaging modality (MRI or CT) and if it is patient level or individual LN assessment. The structure of the "Current state-of-the-art" section is to go through each separate study in detail and since there are a low number of studies we have mentioned all of them explicitly in the text to give a full picture of the existing knowledge base.

We have also included Figure 3 which details a recommended study design based on the best components of the existing approaches. It is included before specific studies are discussed to provide a reference point for readers.

19. *Unify the numbers of digits below zero within tables or manuscript.*

Response:

This has been standardised now.

20. *I guess this journal does not allow using citations in the middle of sentence or as words. Please refer author's instruction once again and correct citations accordingly.*

Response:

We've removed all the instances where it uses the citation as a word in the text, it appears from previous articles that it is possible to cite in the middle of a sentence

21. *Results are too lengthy. Please reduce the length. (13.5 pages -> 7-8 pages)*

Response:

We agree the Results was quite lengthy, we have been through it thoroughly to remove sentences which do not add anything and to simplify the phrasing where possible to make it generally less verbose. In total, we have cut around 700 words from the Results and the main text it is now 10.5 pages. We feel that reducing further would impact the completeness of the results.

22. *Some of the part in results can be moved to discussion part. Discussion is relatively short. Authors pointed out common limitations of previous studies but did not properly suggest what should be included or concerned for better study design. Appropriate number of the data, appropriate way of annotation and setting up ground truth in different settings. Authors mentioned one good study but it is not enough. Authors can do better by suggesting better study design for future studies.*

Response:

Thanks for this guidance we have now shortened the Results and expanded the Discussion. We have added a new paragraph (the 2nd last in the Discussion) to provide insights gained from this review and in that we recommend a more robust study design. These include: a discussion of the current architectures being used and suggesting some other techniques, recommending patient level studies, recommending using automatic segmentation methods based on expert annotations to identify lymph nodes, indicating uncertain predictions for human review and improved validation and explainability to increase clinical transferability. However, note that we have not recommended a dataset size as it has been repeatedly demonstrated that larger datasets are better in computer vision, and it is closely linked to the model complexity.

23. *There are some awkward expression or incomplete sentences throughout the manuscript. please check manuscript carefully and revise English once again.*

Response:

We have been over the manuscript a few times to ensure complete sentences, we corrected grammatical errors and have changed the awkward wording in some areas.

24. *Please cite these references if appropriate in the relevant area.*
    DOI: 10.3390/cancers14112590
    DOI: 10.1093/bib/bbad151
    DOI: 10.3390/cancers12071884

Response:

Thank you for sharing these well structured systematic reviews. We have cited the review on AI for colorectal cancer pathology (https://doi.org/10.3390/cancers12071884) in the introduction to provide evidence for the state-of-the-art performance of AI in colorectal cancer imaging.
